# Localisation musculaire rare d’une tuberculose mimant une tumeur cervicale : à propos d’un cas au Togo

**DOI:** 10.48327/mtsi.v6i1.2026.831

**Published:** 2026-03-18

**Authors:** Essobozou Plaodezina PEGBESSOU, Winga FOMA, Gérémie ANANIDJIN, Essobiziou AMANA

**Affiliations:** 1 Service d’ORL et de chirurgie cervico-faciale du Centre hospitalier régional Lomé-commune, Togo; 2 Service d’ORL et de chirurgie cervico-maxillo-faciale du CHU Sylvanus Olympio de Lomé, Togo

**Keywords:** Tuberculose, Pyomyosite, Sternocléidomastoïdien, GeneXpert, Togo, Afrique subsaharienne, Tuberculosis, Pyomyositis, Sternocleidomastoid, GeneXpert, Togo, Sub-Saharan Africa

## Abstract

**Objectif:**

La révélation d’une tuberculose par une localisation apparemment isolée dans le muscle sternocléidomastoïdien est exceptionnelle. Elle peut prêter à confusion avec une tumeur cervicale. Nous rapportons un cas avec ses particularités diagnostiques et thérapeutiques.

**Observation:**

Patient immunocompétent présentant une tuméfaction cervicale antérolatérale droite faisant suspecter une tumeur de la thyroïde. Les explorations paracliniques ont permis de découvrir une tuberculose du muscle sternocléidomastoïdien droit et un foyer pulmonaire latent homolatéral. L’évolution sous traitement médical a été favorable.

**Conclusion:**

La tuberculose musculaire au niveau cervical peut mimer une tumeur cervicale.

## Abstract (en)

**Objective:**

The detection of tuberculosis in an apparently isolated location, such as the sternocleidomastoid muscle, is exceptional. It can be mistaken for a cervical tumor. This report presents a case with its diagnostic and therapeutic particularities.

**Observation:**

An immunocompetent patient presented with right anterolateral cervical swelling, which was suggestive of a thyroid tumor. Paraclinical investigations revealed tuberculosis of the right sternocleidomastoid muscle and a latent homolateral pulmonary focus. The patient responded well to medical treatment.

**Conclusion:**

Muscular tuberculosis in the cervical region can mimic a cervical tumor.

## Introduction

La tuberculose reste une pathologie endémique dans les pays en voie de développement. La localisation préférentielle est pulmonaire. Au Togo, la fréquence des localisations extra-pulmonaires en 2020 était de 12 % [7]. La révélation par une localisation musculaire cervicale est rare. Nous rapportons un cas de localisation au muscle sternocléidomastoïdien (SCM) droit ayant permis de découvrir un foyer pulmonaire latent homolatéral. Dans un contexte endémique, la pyomyosite tuberculeuse dans cette localisation doit être considérée comme un diagnostic différentiel des tumeurs cervicales.

## Observation

**Figure 1:**
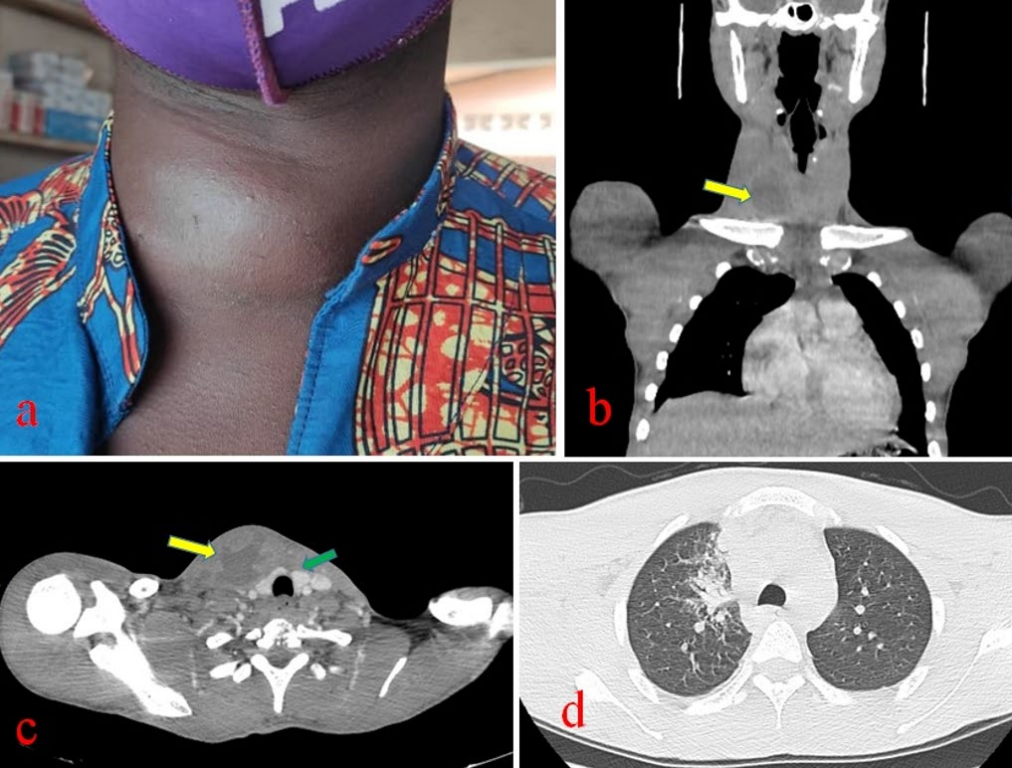
Aspects clinique et scanographique des lésions. a : Aspect clinique de la tuméfaction. b et c : Coupes scanographiques montrant la pyomyosite aux dépens du SCM droit (flèche en jaune) et la thyroïde d’aspect normal (flèche en vert). d : Coupe scanographique montrant le foyer pulmonaire droit.

**Figure 2:**
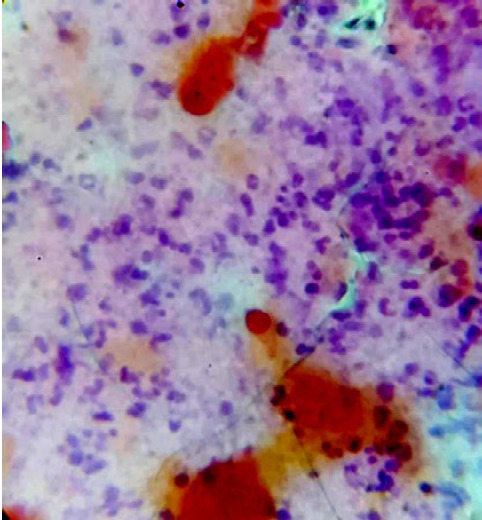
Aspect microscopique de la cytoponction. À noter sur un fond nécrotique des cellules inflammatoires polymorphes à prédominance de lymphocytes (avec macrophages et polynucléaires neutrophiles) orientant en premier vers une étiologie tuberculeuse. MGG; x100

Ce patient de 27 ans a consulté pour une tuméfaction cervicale antérolatérale droite non douloureuse évoluant depuis trois semaines sans notion de fièvre, de toux, de dysthyroïdie ou de douleur thoracique. Il n’avait pas d’antécédent pathologique particulier, ni de notion de contage tuberculeux ou de traumatisme cervical. L’état général était bon. L’examen physique permettait de noter une tuméfaction cervicale antérolatérale droite de six centimètres de grand axe transversal, ferme, peu douloureuse, de contours réguliers, fixée au plan profond et dont le pôle inférieur semblait plonger dans le thorax (Fig. 1a). Il n’y avait pas d’adénopathie cervicale. L’examen des muqueuses ORL était normal. L’hypothèse d’une tumeur plongeante de la thyroïde était évoquée. Une tomodensitométrie (TDM) cervico-thoracique a été réalisée dans le but d’étudier la nature et les rapports de cette masse. Elle a permis de préciser qu’il s’agissait d’une formation ovalaire mal limitée, mixte, à composante tissulaire et liquidienne, développée aux dépens du muscle SCM, se rehaussant discrètement après injection de produit de contraste, et refoulant la thyroïde qui était d’aspect normal. Elle était associée à des adénopathies centimétriques jugulo-carotidiennes droites et médiastinales hautes droites. On constatait une pneumopathie alvéolaire apico-dorsale droite (Fig. 1b, 1c et 1d). Une cytoponction de la tuméfaction a ramené un liquide purulent non fétide. Son étude cytologique a montré un infiltrat lymphocytaire avec des macrophages et des polynucléaires neutrophiles, compatible avec une tuberculose (Fig. 2). Le GeneXpert du liquide de ponction a permis la détection de *Mycobacterium* du complexe *tuberculosis*, souche sensible à la rifampicine. Le diagnostic de tuberculose à localisation musculaire et pulmonaire droit a été retenu.

Le patient a bénéficié d’une quadrithérapie antituberculeuse par rifampicine, isoniazide, pyrazinamide et éthambutol pendant deux mois, puis d’une bithérapie par rifampicine et isoniazide pendant quatre mois. L’évolution a été favorable. Il n’y a pas eu de récidive après quatre ans de recul.

## Discussion

Les formes extra-pulmonaires de la tuberculose sont essentiellement représentées par les atteintes ganglionnaires, osseuses, articulaires, méningées, et génitales. Des collections dans les tissus conjonctifs notamment sous-cutanés sont parfois rencontrées. Les atteintes musculaires sont exceptionnelles. De rares cas d’atteinte isolée ou associée du muscle SCM ont été rapportés dans la littérature [2,8]. Le muscle strié ne serait pas favorable au développement de *M. tuberculosis* en raison de sa faible teneur en oxygène, sa forte concentration en acide lactique et sa pauvreté en tissu réticuloendothélial.

L’atteinte d’un muscle survient habituellement par extension d’un foyer osseux ou articulaire, par inoculation directe ou par dissémination hématogène ou lymphatique [4,5,8]. L’atteinte musculaire primitive isolée est extrêmement rare et survient le plus souvent chez le sujet immunodéprimé [2,4]. Dans le cas rapporté, l’atteinte musculaire serait secondaire à une contamination par voie hématogène ou lymphatique à partir du foyer pulmonaire droit qui est resté latent. La radiographie pulmonaire peut paraître normale dans les formes latentes de la tuberculose [1]. La TDM est plus sensible que la radiographie standard dans la détection des lésions infracliniques de la tuberculose primaire, et participe ainsi à son diagnostic précoce comme le montre notre observation [1,8]. Dans les localisations musculaires, l’IRM est l’examen de choix car il explore mieux les tissus mous [4]. L’échographie garde son intérêt dans ce type d’atteinte. La cytoponction permet de faire des explorations histologiques et/ou bactériologiques des tuméfactions cervicales. Le diagnostic de certitude de la tuberculose est bactériologique et/ou histologique. Dans les formes non bacillifères de la tuberculose (formes extra-pulmonaires), le GeneXpert, technique de biologie moléculaire, est d’un intérêt majeur dans le diagnostic précoce et dans la détection des souches résistantes à la rifampicine [3]. Même dans la forme pulmonaire, ce test a une plus grande sensibilité que la bacilloscopie [3].

La localisation sternocléidomastoïdienne de la tuberculose peut prêter à confusion avec les tumeurs cervicales, posant ainsi un problème de diagnostic différentiel. La clinique seule laisse dans l’incertitude du fait de la rareté de cette localisation et de l’absence de signes cliniques spécifiques, d’où l’intérêt d’examens paracliniques appropriés [2,8].

**Figure 3:**
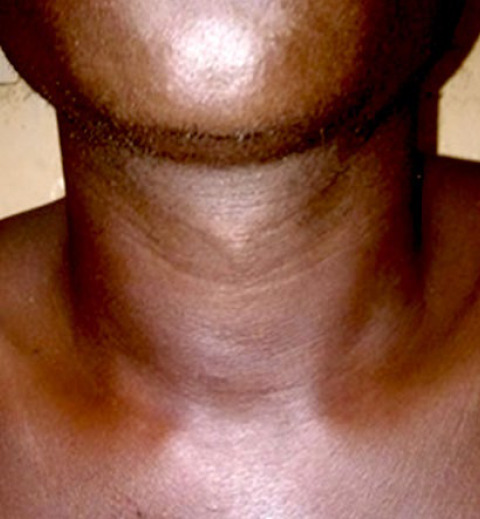
Aspect du cou en fin de traitement

Le traitement des localisations musculaires est médico-chirurgical par quadruple antibiothérapie antituberculeuse et par drainage de l’abcès [6]. Notre patient a bénéficié uniquement du traitement médical sur une période de six mois avec une bonne évolution (Fig. 3), ce qui prouve son efficacité lorsque le diagnostic est précoce.

## Conclusion

La tuberculose musculaire est une affection rare, qui doit être évoquée dans un contexte endémique. Sa localisation cervicale doit être considérée comme un diagnostic différentiel des tumeurs cervicales. Un diagnostic précoce permet un traitement médical approprié et la prévention des complications sans avoir recours à la chirurgie.

## Consentement éclairé

Nous avons obtenu le consentement oral du patient.

## Contribution des auteurs

Conception du rapport de cas : Essobozou Plaodezina PEGBESSOU, Winga FOMA Prise en charge diagnostique et thérapeutique du patient, rédaction, relecture et validation du document : Winga FOMA, Gérémie ANANIDJIN Rédaction, relecture et validation du document : Essobozou Plaodezina PEGBESSOU, Essobiziou AMANA

## Déclaration de liens d’intérêt

Aucun lien d’intérêt n’a été déclaré.

## References

[1] Beigelman C, Brauner M, Soussan M, Arrigoni PP, Brillet PY, Baunin C, Beigelman C, Brauner M, Carette MF, Debray MP, Ducou le Pointe H (Mai 2013). Tuberculose pulmonaire et mycobacterioses atypiques. Imagerie Thoracique.

[2] Boussetta N, Mettoui L, Guediche NH, Abid R, Ajili F, Batikh R, Louzir B, Gharsallah I, Othmani S (2015). La tuberculose musculaire isolée. La Revue de médecine interne..

[3] Diop SA, Massaly A, Ka D, Manga NM, Fortes-Déguénonvo L, Ndour CT, Cisse VMP, Seydi M (2016). Utilisation du test GeneXpert pour le diagnostic de la tuberculose au service des maladies infectieuses du CHNU de Fann. Pan Afr Med J..

[4] Elleuch E, Ammari L, Kilani B, Benaissa HT, Ghoubantini A, Kanoun F, Abdelmalek R, Chaabane TB (2011). Un cas de pyomyosite tuberculeuse. Revue tunisienne d’infectiologie. Avril.

[5] Hayoun S, Ouazzani HE, Habibi B, Belhabib S, Souhi H, Rhorfi IA, Abid A (2017 May 18). Tuméfaction du muscle pectoral révélant une tuberculose musculaire isolée. Pan Afr Med J..

[6] Kim YJ, Jeon HJ, Kim CH, Park JY, Jung TH, Lee EB, Park TI, Jeon KN, Jung CY, Cha SI (2009). Chestwall tuberculosis: clinical features and treatment outcomes. Tuberc Respir Dis..

[7] Organisation mondiale de la santé (OMS) (2021). Rapport sur la tuberculose dans le monde 2021.

[8] Priya M, Angral S, Sood R, Malhotra M, Bhardwaj A, Gupta MK (2020). Primary tuberculous myositis of sternocleidomastoid and anterior scalene muscle: A report of three cases. IP Indian J Anat Surg Head Neck Brain..

