# Supplementary material for: Rare muscular localization of tuberculosis mimicking a cervical tumor: a case report from Togo
Source: Med Trop Sante Int. 2026 Mar 18;6(1):mtsi.v6i1.2026.831. [Article in French] doi: 10.48327/mtsi.v6i1.2026.831 (PMC13216898; doi:10.48327/mtsi.v6i1.2026.831)
Supplement: Related texts

# Research article: Rare muscular localization of tuberculosis mimicking a cervical tumor: a case report from Togo

## Introduction

Tuberculosis is still endemic in developing countries. It primarily affects the lungs. In Togo, extrapulmonary localization occurred in 12% of cases in 2020 [7]. Detection through cervical muscle localization is rare. Here, we present a case of localization in the right sternocleidomastoid (SCM) muscle that revealed a latent pulmonary focus on the same side. In an endemic setting, tuberculous pyomyositis in this location should be considered in the differential diagnosis of cervical tumors.

## Observation

This 27-year-old patient presented with painless right anterolateral cervical swelling that had developed over three weeks. There was no associated fever, cough, dysthyroidism, or chest pain. The patient had no significant medical history and no history of tuberculosis infection or cervical trauma. He was in good general health. Physical examination revealed a firm, slightly painful swelling on the right anterolateral side of the neck. The swelling measured 6 cm in transverse diameter and had regular contours. It was fixed to the deep plane, and the lower pole appeared to extend into the thorax (Fig. 1a). There was no cervical lymphadenopathy. Examination of the ENT mucosa was normal. The hypothesis of a plunging thyroid tumor was raised.

**Figure 1:** Clinical and CT scan images of the lesions. a. Clinical appearance of the swelling. b. and c. CT scan images showing pyomyositis affecting the right SCM muscle (yellow arrow) and a normal-appearing thyroid gland (green arrow). d. CT scan image showing the right lung lesion.

A cervical-thoracic computed tomography (CT) scan was performed to study the nature and relationships of this mass. The scan revealed a poorly defined, mixed, oval formation with tissue and fluid components that developed at the expense of the SCM muscle. The formation enhanced slightly after contrast injection and displaced the normal-appearing thyroid. The mass was associated with centimeter-sized right jugular-carotid and upper right mediastinal lymphadenopathy. Right apico-dorsal alveolar pneumonia was also observed (Figs. 1b, 1c, and 1d). Fine-needle aspiration of the swelling yielded a purulent, non-foul-smelling fluid. Cytological examination showed a lymphocytic infiltrate with macrophages and neutrophils, which is consistent with tuberculosis (Fig. 2). A GeneXpert analysis of the puncture fluid detected a strain of *Mycobacterium tuberculosis* complex that is sensitive to rifampicin. This confirmed the diagnosis of tuberculosis in the right muscle and lung.

**Figure 2:** Microscopic appearance of fine needle aspiration. Note the necrotic background of polymorphonuclear inflammatory cells, predominantly lymphocytes, as well as macrophages and neutrophils. This initially suggests a tuberculous etiology. MGG; x100

The patient received a four-drug antituberculosis regimen consisting of rifampicin, isoniazid, pyrazinamide, and ethambutol for two months. This was followed by a two-drug regimen consisting of rifampicin and isoniazid for four months. The outcome was favorable. There has been no recurrence after four years of follow-up.

## Discussion

Extra-pulmonary forms of tuberculosis primarily affect the lymph nodes, bones, joints, meninges, and genitals. Connective tissue collections, particularly subcutaneous tissue, are sometimes encountered.

Muscle involvement is exceptional. There have been rare cases reported in the literature of isolated or associated involvement of the SCM muscle [2,8]. Striated muscle is not conducive to the development of *M. tuberculosis* due to its low oxygen content, high lactic acid concentration, and lack of reticuloendothelial tissue.

Muscle involvement typically occurs via extension from a bone or joint focus, direct inoculation, or hematogenous or lymphatic dissemination [4,5,8]. Primary muscle involvement that is isolated and occurs without other organ involvement is extremely rare and most often occurs in immunocompromised individuals [2,4]. In the reported case, the muscle involvement appears to be secondary to hematogenous or lymphatic spread from a latent pulmonary focus on the right side. Chest X-rays may appear normal in latent tuberculosis [1]. CT scans are more sensitive than standard radiography in detecting subclinical lesions of primary tuberculosis, thus contributing to early diagnosis, as demonstrated by our observations [1,8]. For muscular locations, MRI is the preferred method because it provides better visualization of soft tissues [4]. Ultrasound remains useful for this type of lesion. Fine needle aspiration allows for a histological and/or bacteriological examination of cervical swellings. The definitive tuberculosis diagnosis is bacteriological and/or histological. In non-bacilliferous forms of tuberculosis (i.e., extrapulmonary forms), the GeneXpert molecular biology technique is of major interest in the early diagnosis of tuberculosis and in detecting rifampicin-resistant strains [3]. This test is even more sensitive than bacilloscopy in the pulmonary form [3].

The sternocleidomastoid location of tuberculosis can be confused with cervical tumors, thus posing a differential diagnosis problem. Clinical presentation alone leaves uncertainty due to the rarity of this location and the absence of specific clinical signs, hence the importance of appropriate paraclinical tests [2,8].

Muscle localization is treated medically and surgically with quadruple anti-tuberculosis antibiotic therapy and abscess drainage [6]. Our patient received only medical treatment over a period of six months with good results (Fig. 3), proving its effectiveness when diagnosed early.

**Figure 3:** Appearance of the neck at the end of treatment

## Conclusion

Muscular tuberculosis is a rare condition that should be considered in endemic areas. Its cervical location should be considered as a differential diagnosis for cervical tumors. Early diagnosis allows for appropriate medical treatment and the prevention of complications without resorting to surgery.

## Informed consent

We obtained the patient’s verbal consent.

## Authors’ contributions

Case report design: Essobozou Plaodezina PEGBESSOU, Winga FOMA

Diagnostic and therapeutic management of the patient, writing, proofreading, and validation of the document: Winga FOMA, Gérémie ANANIDJIN Writing, proofreading, and validation of the document: Essobozou Plaodezina PEGBESSOU, Essobiziou AMANA

## Conflicts of interest

The authors declare no conflicts of interest
